# Multi-classifier fusion based on belief-value for the diagnosis of autism spectrum disorder

**DOI:** 10.3389/fnhum.2023.1257987

**Published:** 2023-11-22

**Authors:** Feng Zhao, Shixin Ye, Mingli Zhang, Ke Lv, Xiaoyan Qiao, Yuan Li, Ning Mao, Yande Ren, Meiying Zhang

**Affiliations:** ^1^School of Computer Science and Technology, Shandong Technology and Business University, Yantai, China; ^2^School of Statistics, Shandong Technology and Business University, Yantai, China; ^3^School of Management Science and Engineering, Shandong Technology and Business University, Yantai, China; ^4^Department of Radiology, Yantai Yuhuangding Hospital, Yantai, China; ^5^Department of Radiology, The Affiliated Hospital of Qingdao University, Qingdao, China; ^6^Qingdao Hospital, University of Health and Rehabilitation Sciences (Qingdao Municipal Hospital), Qingdao, China

**Keywords:** resting-state functional magnetic resonance imaging (rs-fMRI), autism spectrum disorder (ASD), belief-value, multi-classifier fusion, feature selection

## Abstract

**Introduction:**

Autism Spectrum Disorder (ASD) has a significant impact on the health of patients, and early diagnosis and treatment are essential to improve their quality of life. Machine learning methods, including multi-classifier fusion, have been widely used for disease diagnosis and prediction with remarkable results. However, current multi-classifier fusion methods lack the ability to measure the belief level of different samples and effectively fuse them jointly.

**Methods:**

To address these issues, a multi-classifier fusion classification framework based on belief-value for ASD diagnosis is proposed in this paper. The belief-value measures the belief level of different samples based on distance information (the output distance of the classifier) and local density information (the weight of the nearest neighbor samples on the test samples), which is more representative than using a single type of information. Then, the complementary relationships between belief-values are captured via a multilayer perceptron (MLP) network for effective fusion of belief-values.

**Results:**

The experimental results demonstrate that the proposed classification framework achieves better performance than a single classifier and confirm that the fusion method used can effectively fuse complementary relationships to achieve accurate diagnosis.

**Discussion:**

Furthermore, the effectiveness of our method has only been validated in the diagnosis of ASD. For future work, we plan to extend this method to the diagnosis of other neuropsychiatric disorders.

## 1 Introduction

Autism spectrum disorder (ASD) is a complex genetically heterogeneous neurological disorder with a high prevalence, often coexisting with other disorders (Hiremath et al., 2021). A recent report from the Centers for Disease Control and Prevention showed that one in every 54 American children aged 8 years has ASD with varying degrees of severity, which creates an enormous socioeconomic burden on society (Eslami et al., 2019; Lord et al., 2020; Maenner et al., 2021). Therefore, early identification and treatment of ASD are of great clinical value (Wang et al., 2019).

Given the importance and complexity of ASD diagnosing, it is essential to find effective and reliable methods that help clinicians diagnose patients. Currently, many researchers use machine learning to assist in the diagnosis of ASD based on neuroimaging and they have achieved promising results (Bone et al., 2015; Nogay and Adeli, 2020; Raj and Masood, 2020; Thabtah and Peebles, 2020). For example, Ahmed et al. (2020) used the raw pixel feature obtained from fMRI data with support vector machines (SVM) to diagnose ASD. Karampasi et al. (2020) used Haralick texture features extracted from resting-state functional magnetic resonance imaging (rs-fMRI) for ASD diagnosis. Vigneshwaran et al. (2015) used regional homogeneity of voxels from MRIs as a feature to diagnose ASD in men. The abovementioned methods only consider a single feature extracted from neuroimaging with a single classifier to assist in diagnosis. However, considering the complexity and heterogeneity of ASD, the limited information expressed by a single feature makes it difficult to provide comprehensive information, and the diagnosis by a single classifier with a single feature will not meet the clinical needs (Kuncheva et al., 2001).

Inspired by the concept of multi-view learning (Li et al., 2022), which uses information from multiple views to enhance an object’s representation, multiple features extracted from neuroimaging with multiple-classifier fusion can be used in a similar way to enhance the representation of subjects (Huang et al., 2019). To overcome the limitations of a single classifier, the multi-classifier fusion has been intensively studied in recent years. In general, multiple features exhibit complementary characteristics in classification. Therefore, if multiple classifiers with different input features are used and their complementary information is effectively fused through fusion methods to obtain the final classification results, the overall performance is expected to be superior to the best performance of a single classifier (Ruta and Gabrys, 2000).

Multi-classifier fusion can be roughly divided into two classes in terms of the type of output generated by each classifier: based on the class labels and based on the prediction probability. *The first class* is generally based on the majority voting principle (Mousavian et al., 2020), which integrates the class labels by the most frequently appeared result in all voting results. Takruri et al. proposed to use a majority voting approach to merge individual predictions with multiple features based on different definitions (Takruri et al., 2016). However, simple majority voting treats each classifier equally without considering the impact of misclassified classifiers, which may lead to a decrease in overall prediction accuracy. Ichinose et al. (2015) used weighted voting to assign weights to different classifiers based on the importance of different features and obtained better classification results. Although the specificity of multiple classifiers was considered, the method suffers from the difficulty of determining classifier weights. Overall, majority voting methods fuse multiple classifiers based only on the class label of each classifier, while ignoring some additional useful information, such as the prediction probability output by the classifiers.

*The second class* is based on prediction probability. Typically, the prediction probability output by the classifiers is used to measure the accuracy of the classifier in predicting the classification assignment (Li and Sethi, 2006). Mathematically, the prediction probability is generally calculated by using the confusion matrix of the classifier output. For instance, Zhao et al. (2023) defined a belief-value (i.e., prediction probability, which is used to measure the belief level that a sample belongs to a certain class) based on the confusion matrix of each classifier, and then linearly fused the belief-values of all different classifiers to achieve improved classification performance. However, there is still a problem in that different input samples have the same belief-value, which is calculated based on the confusion matrix. The accuracy of a sample belonging to a class is supposed to be different for different samples due to their different characteristics, thus different samples should get different belief-values. In addition, a linear fusion of the belief-value in multi-classifier fusion ignores the non-linear relationship between classifiers, which indirectly affects the fusion of complementary information and the reliability of the overall classification.

Furthermore, in order to reasonably evaluate the belief-values for different samples, the definition of belief-value for different samples was investigated. Currently, most classifiers reflect the difference in belief-value of different samples based on the prediction probability of the classifier. For example, Zhao et al. (2020) used the distance information (i.e., the output distance of SVM) in SVM as a belief-value to measure the prediction probability for a single feature belonging to the sample, and achieved accurate diagnosis of ASD by further fusing the belief-value of different features. However, due to the imbalance of sample distribution and inappropriate classifier selection, the classifier could not correctly classify all samples, and there was a case where the misclassified samples output the wrong belief-value, with inaccurate belief-value of samples further affecting the accuracy of multi-classifier fusion. Another belief-value is defined based on local density information, Aslandogan and Mahajani (2004) used the nearest neighbor samples to calculate the local density information of each sample, and the belief-values of the samples by averaging the nearest neighbor sample weights. However, its weight definition leads to the appearance of anomalous weights, which affects the accuracy of belief-value. Based on the current study, a reasonable definition of the belief-value of the sample through prior probability deserves further exploration. While the two classes of multi-classifier fusion methods mentioned above have made some progress (Rohlfing et al., 2004; Ranawana and Palade, 2006; Prasad et al., 2008), they still have two limitations. (1) The construction of belief-value based on predicted probability for different samples is not reasonable and (2) fusion methods that fuse belief-value fail to better capture complementary information.

**FIGURE 1:**
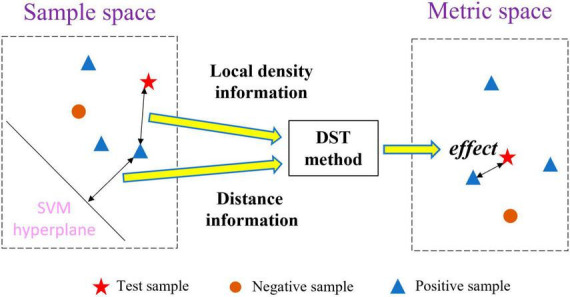
Space transformation by the DST method.

To address the abovementioned problems, we propose a new multi-classifier fusion classification framework based on belief-value for identifying ASD. The belief-value is the expectation value of the “effect” from all the nearest neighbor samples on the test sample in the metric space, which is transformed from the sample space with a certain feature by the distribution-based spatial transformation (DST) method. Figure 1 shows the transformation process of the DST method. The DST method combines distance information and local density information to transform the sample space into the metric space, which effectively combines the information from both perspectives to enhance the representation of the belief-value. Further, the belief-value of the test sample is calculated by averaging the “effect” of all the nearest neighbor samples. Finally, the belief-values from the sample space with different features are fused by a multilayer perceptron (MLP) network to capture the non-linear relationship between the different belief-values and output the final classification result.

Our work has made the following main contributions: (1) We proposed a new belief-value based on distance information and local density information, which can measure the belief level of different samples (such as different subjects) and has a more reasonable representation for belief-value. (2) We captured the non-linear relationship between the belief-value of multiple classifiers through an MLP network, which achieves better fusion of complementary information between multi-classifiers compared to linear fusion methods. Experimental results demonstrate that our classification framework achieves better performance than single classifiers, and reasonable belief-value definition and effective fusion methods are the keys to the classification framework.

## 2 Materials and methods

### 2.1 Data acquisition

The data used in this article comes from the Autism Brain Imaging Data Exchange (ABIDE) database, which is composed of 17 imaging sites worldwide (Alcaraz and Rieta, 2010; Di Martino et al., 2014). To address data heterogeneity, we selected the rs-fMRI data from the NUY site, which is the largest sample size to test the feasibility of our proposed method. We include rs-fMRI scanning data from 45 patients with ASD and 47 normal control (NC) subjects, with ages ranging from 7 to 15 years and no excessive head movements in any three directions, displacement less than 1.5 mm, or angular rotation less than 1.5°. The detailed demographic information of these subjects is summarized in Table 1, as pointed out by previous research (Wee et al., 2016; Zhao et al., 2020). No significant differences (*p* > 0.05) in age, sex, IQ, diagnostic interview, or diagnostic observation were found between the two groups.

**TABLE 1:** Demographic information of the subjects.

Characteristic	ASD	NC	p-Value
Sex (M/F)	36/9	36/11	0.6923^a^
Age (mean ±SD)	11.1 ±2.3	11.0 ±2.3	0.773^b^
FIQ (mean ±SD)	106.8 ±17.4	113.3 ±14.1	0.0510^b^
ADI-R (mean ±SD)	32.2 ±14.3^c^	–	–
ADOS (mean ±SD)	13.7 ±5.0	–	–
FD (mm) (mean ±SD)	0.14 ±0.05	0.15 ±0.07	0.36^b^

M, male; F, female; FIQ, Full Intelligence Quotient; ADI-R, Autism Diagnostic Interview-Revised; ADOS, autism diagnostic observation schedule; SD, standard deviation.

^a^The *p*-value was obtained by a χ^2^-test.

^b^The *p*-value was obtained by a two-sample two-tailed *t*-test.

^c^Two patients did not have the ADI-R score.

Specifically, the rs-fMRI data were acquired using a 3.0 T Siemens Allegra scanner. During the resting-state scan, participants were instructed to keep their eyes open and fixate on a white cross presented on a black screen. The scan lasted for 6 min, resulting in the acquisition of 180 volumes of EPI images [repetition time (TR)/echo time (TE) = 2,000/15 ms, flip angle = 90°, 33 slices, slice thickness = 4 mm, imaging matrix = 64 × 64].

### 2.2 Data preprocessing

Preprocessing of the data was performed using the Analysis of Functional NeuroImages (AFNI) software (Cox, 1996). The preprocessing steps included discarding the first 10 volumes of the R-fMRI data, spatial smoothing using a Gaussian kernel with a full width at half maximum (FWHM) of 6 mm, signal detrending, band-pass filtering (0.005–0.1 Hz), regression of nuisance signals (ventricle, white matter, and global signals), and normalization to the Montreal Neurological Institute (MNI) space with a voxel resolution of 3 mm × 3 mm × 3 mm. To mitigate the effects of head motion, six head motion signals were regressed prior to computing functional connectivity (Murdaugh et al., 2012; Satterthwaite et al., 2013; Yan et al., 2013; Washington et al., 2014; Leung et al., 2015; Ray et al., 2015; Urbain et al., 2016; Reinhart and Nguyen, 2019). The Automated Anatomical Labeling (AAL) maps were used to divide the brain into 116 regions of interest (ROIs). We calculated the mean value of the rs-fMRI time series for each ROI, which resulted in a data matrix *X* ∈ *R*^170 × 116^, where 170 represents the total number of time images and 116 represents the total number of brain ROIs, which was used in experiments.

### 2.3 Classification framework

**FIGURE 2:**
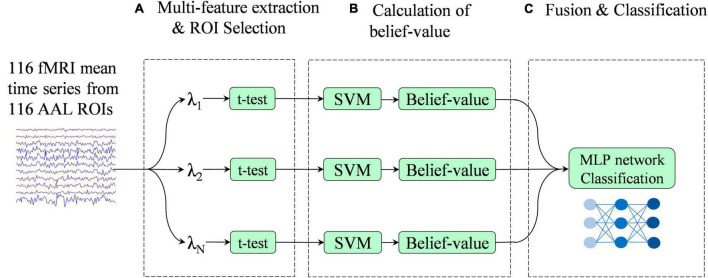
Pipeline of the proposed classification framework. This framework consists of three stages: **(A)** multi-feature extraction and region of interest (ROI) selection, **(B)** calculation of belief-value, and **(C)** fusion and classification.

Figure 2 illustrates the overview of the proposed multi-classifier fusion classification framework for identifying ASD.

### 2.4 Multi-feature extraction and ROI selection

The researchers achieved favorable classification results based on the spatio-temporal features as well as the non-linear dynamics features extracted from the rs-fMRI series data (Mao et al., 2019; Li et al., 2021a,b). The spatio-temporal features mainly include time-domain and frequency-domain features. Time-domain features refer to the description and analysis of the characteristics of rs-fMRI series data in the time dimension, such as the mean, variance, kurtosis, skewness, etc. Time-domain features can reflect the change of data in time and, therefore, can describe the dynamic characteristics of data, such as the trend, periodicity, and rate of change of data. Frequency-domain features refer to the features in the frequency-domain dimension obtained after the frequency-domain transformation of the rs-fMRI series data, such as the model of variational mode decomposition (VMD) (Dragomiretskiy and Zosso, 2013). Frequency-domain features can reflect the distribution of data in frequency and, therefore, can describe the static characteristics of the data. In addition, the non-linear dynamics feature is also an important description method for rs-fMRI series data, where entropy is a non-linear dynamics feature that can be used to describe the complexity, information quantity, and randomness of the series.

In this research article, the time-domain, frequency-domain, and entropy features of the rs-fMRI series data are extracted from the rs-fMRI series data. Time-domain features include (1) mean, (2) variance, (3) kurtosis, and (4) skewness of the series data.

The frequency-domain features include (1) the modes decomposed by the VMD, and (2) the amplitude of low-frequency fluctuations (ALFF) (Zou et al., 2008). In the VMD, the series data is decomposed into multiple intrinsic mode functions (IMFs), each of which represents a frequency component in the series, and each IMF can be used to describe the vibrational modes and characteristics of the original signal in a specific frequency range. The ALFF reflects the average strength in the low-frequency part of each rs-fMRI series data.

The sample entropy (Alcaraz and Rieta, 2010) is used as a feature in the entropy feature, which is a statistic used to analyze a series to assess its complexity and irregularity. For a subject’s rs-fMRI series data matrix *X* ∈ *R^a × b^*, where *a* represents the total volume of time images and *b* represents the total number of brain ROIs, we extract features from the *X* by the abovementioned feature types, and all features for a subject are expressed as $\lambda ={\left\{\lambda_{i}\right\}}_{i=1}^{N}\in R^{1\times b}$, where *N* is the number of feature types that are used.

The ROI selection performs a two-sample *t*-test between NC subjects and ASD subjects, with ROIs with *p*-values of less than a certain threshold being preserved. The equation λ=′{λ′i}i=1N∈R1×h denotes all features after ROI selection, where *h* is the number of ROIs by ROI selection.

### 2.5 The output probability of SVM

**FIGURE 3:**
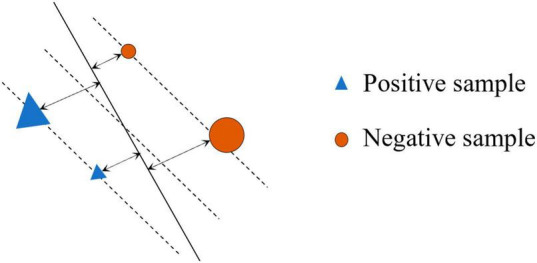
Sample with SVM margin.

Equation $\text{A}\,\left(x\right)={\left\{x_{i}\right\}}_{i=1}^{n+m}$ denotes all training samples in the sample space, where *n* and *m* are the number of training samples on both sides of the SVM hyperplane. The A(*x*) is divided into two subsets by SVM hyperplane, namely, positive train points and negative train points, denoted as $A^{+}\,\left(x\right)={\left\{x_{i^{+}}\right\}}_{i^{+}=1}^{n}$ and $A^{-}\,\left(x\right)={\left\{x_{i^{-}}\right\}}_{i^{-}=1}^{m}$, respectively. With the introduction of the hyperplane, each sample has a new property, namely, the output probability of SVM, that is, probabilistic representation for the geometric distance of the sample from the hyperplane (i.e., SVM-margin). SVM-margin for point *x_i_* in the sample space is the signed distance between *x_i_* and the decision boundary, ranging from to + ∞. A positive SVM margin for *x_i_* indicates that *x*_*i*_ is predicted to belong to that positive class, and vice versa. For a sample point (*x*_*i*_,*y*_*i*_), where *y_i_* is the label for *x_i_*, the SVM margin is the geometric interval *r*_*i*_ of the hyperplane about the (*x*_*i*_,*y*_*i*_), as follows:


(1) $$r_{i}=\frac{y_{i}\,\left(\left(w*x_{i}\right)+b\right)}{{||w||}^{2}}$$

where ${||w||}^{2}$ is the *L*_2_-norm for w . Figure 3 shows the SVM margin in the sample space, the triangle and circle (△ and ○) stand for the two types of points to be separated. The area occupied by the two figures stands for the corresponding SVM margin of the sample point.

In general, the distance of a point from the hyperplane is the SVM margin that can indicate the degree of certainty for the classification prediction. Furthermore, the SVM margin can be transformed into the form of probabilities, as follows:


(2) $$s_{i}=\frac{1}{1+e\,x\,p\,\left(-\left|r_{i}\right|\right)}\times s\,i\,g\,n\,\left(r_{i}\right)$$

where *r*_*i*_ is the SVM margin for *x_i_*, and *Sign* is the sign function that returns the sign of its input value (i.e., {−1,1}). The *s*_*i*_ is the output probability of SVM, which is a value with a sign and indicates the classification result of SVM for *x_i_*.

### 2.6 Belief-value

Before continuing, a note on mathematical notations is given as follows. The belief-value (denoted as B−V) for test sample *x* is based on the local density in the sample space with the SVM hyperplane. The *x* belongs to a subset of A(*x*), *A*^+^(*x*) or *A*^−^(*x*). Let $\text{NN}={\left\{n_{j}\right\}}_{j=1}^{p}$ denote all samples in the subset except *x*, and consider ***NN*** as the nearest neighbor samples of *x*, where *p* is denoted as the number of nearest neighbor samples. The distance between *n_j_* and *x* is denoted as *d_j_*, which is in the form of Euclidean distance or Mahalanobis distance. Let $\text{D}={\left\{d_{j}\right\}}_{j=1}^{p}$ denote the distance between *x* and all nearest neighbor samples in ***NN***. According to Eq. 2, the *s_j_* for *n_j_* is derived from the distance between *n_j_* and the hyperplane. Let $\text{S}={\left\{s_{j}\right\}}_{j=1}^{p}$ denote the output probability of SVM for all nearest neighbor samples in ***NN***.

**FIGURE 4:**
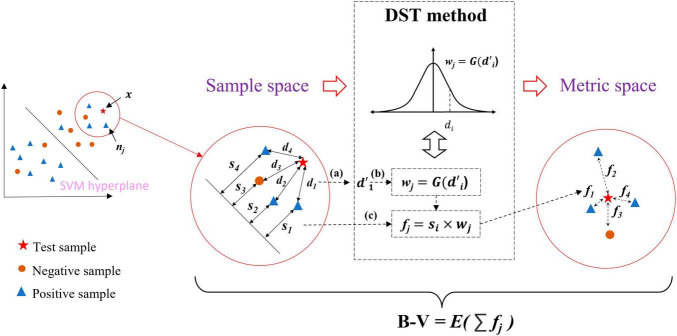
Overview of belief-value calculations for the test sample. (a) Normalization process of distance. (b) Conversion of distance into weight. (c) Definition of “effect”.

Figure 4 briefly illustrates the calculation of the B−V for the *x*. The distance representation obtained from the two information from two perspectives (i.e., *d_j_* and *s_j_*) transforms the sample space into the metric space by the DST method, and the belief-value for *x* is calculated based on the expectation of the “effect” from nearest neighbor samples at ***NN*** on the *x* in the metric space. The following details the calculation process of B−V.

First, the *d_j_* and *s*_*j*_ in the sample space were obtained; it is particular that the distance metric for *d_j_* in the sample space used the Mahalanobis distance (i.e., Eq. 4), which takes into account the covariance structure of the data and the correlation between the variables. Then *d_j_* in **D** was normalized to d′jbased on Eq. 4, where μ and σ are the mean and variance of **D**. Figure 4a is the normalized process.


(3) $$d_{j}\,\left(x,x_{j}\right)=\sqrt{{\left(x-x_{j}\right)}^{T}\,\Sigma^{-1}\,\left(x-x_{j}\right)}$$

(4) d′j=dj-μσ

In the sample space, the SVM has made a classification of *x*. However, there exists such a situation: assuming that *x* is to be classified, the SVM makes a classification of *x* and assigns it a label *y*_*svm*_ = *c*, where *c* ∈ {−1, 1}, there is case where *y*_*svm*_ is not assigned the correct label. Inspired by the concept of utilizing local density information to classify samples, the correct classification result of *x* can be derived from the local density information. Therefore, the DST method is utilized to transform the sample space into the metric space where *x* is reclassified by using the nearest neighbor information in the metric space.

Typically, the way to classify samples by local density information is to use a weight function. The d′j is considered to be transformed into weight *w*_*j*_ by using Eq. 5. The weight is multiplied by the label of *n_j_* to get an effect value of *n_j_* on *x*. Averaging all the effect values of ***NN*** leads to the classification of *x*.


(5) wj⁢(d′j)=1d′j⁢d′j≠0

In order to solve the problem that the weight may be unreasonable due to the very large exception values, we consider the Gaussian probability density function for the d′j between the *x* and the *x_j_* as the weight function, with the assumption that d′j follows Gaussian distribution. Figure 4b and Eqs 6, 7 show the calculation process of distance d′j to weights *w_j_*:


(6) G⁢(d′j)=1σ⁢2⁢π⁢e⁢x⁢p⁢(-d′j⁢(x,xj)22⁢σ2)

(7) wj=G⁢(d′j)

where G⁢(d′j) is the one-dimensional Gaussian probability density function with a mean of 0 and variance of 1.

Due to the *s_j_* as the function of measuring the belief level of *x_j_*, considering this effect on the *x*, we multiply the *s_j_* with the *w*_*j*_ to obtain the “effect” of *x_j_* on *x*. The “effect” is defined as the distance between *x_j_* and *x* in the metric space and is denoted as *f_j_*, which is shown in Figure 4c and Eq. 8. It is a symbolic value for *f_j_* since *s_j_* contains the label information from the SVM output. The *f_j_* represents the classification contribution of *n_j_* to *x* in the metric space.


(8) $$f_{j}=w_{j}*s_{j}$$

The contribution of *s_j_* to *f_j_* can be considered as the information from the perspective of distance is employed. Then the calculation process of B-V can be considered as the local density information is employed, which calculates the expectation of all *f_j_* of *n_j_* by Eq.9:


(9) $$B-V=E\,\left(\sum_{j=1}^{n}f_{j}\right)$$

where B−V is a sign score, and its sign can indicate the classification to which the sample belongs. Therefore, the calculation of the B−V is the process of classifying the sample based on the corresponding feature; the process of computing B−V can be considered as a classifier and the classification result is given by the following equation:


(10) $$\hat{y}=S\,i\,g\,n\,\left(\text{B}-V\right)$$

where $\hat{y}\in \left\{\hat{-1},1\right\}$ is the final classification result. The B−V is the property that measures the belief level for *x* from the perspective of distance and the local density. According to the above, λ′ denotes all features of a subject after ROI selection; the multi-classifier is set independently for all features, and the multi-classifier outputs multiple B−V, denoted as ${\left\{\text{B}-V_{i}\right\}}_{i=1}^{N}$, where *N* is the types of features.

### 2.7 Fusion method

Fusion methods combine the output results of multi-classifiers to improve classification performance and accuracy (Kittler et al., 1998; Mangai et al., 2010; Giannakakis et al., 2017). The basic idea of the classifier fusion method is that by combining the decision results of multi-classifiers, the shortcomings of a single classifier can be compensated and the performance and robustness of the classifier can be improved. In this research article, ${\left\{\text{B}-V_{i}\right\}}_{i=1}^{N}$ as decision results of classifiers were fused by the fusion method.

Three fusion methods were used to improve the classification performance by fusing the ${\left\{\text{B}-V_{i}\right\}}_{i=1}^{N}$ of multi-classifiers: majority voting, linear SVM, and multilayer perceptron (MLP) networks. Majority voting is a common multi-classifier fusion method that votes on the predictions of multiple classifiers and selects the class with the most votes as the final classification result. The formula for majority voting is as follows:


(11) $$\hat{y}=S\,i\,g\,n\,\left(\Sigma_{j=1}^{N}\,S\,i\,g\,n\,\left(\text{B}-V_{j}\right)\right)$$

where $\hat{y}$ is the final classification result, *N* is the number of classifiers, and B−V_*j*_ is the belief-value by the *j*-th classifier. In majority voting, for each class, we count the number of times it is predicted by all the classifiers. The class with the highest count is selected as the final classification result.

Linear SVM performs well in handling high-dimensional and small-sample data, and can effectively solve linearly separable problems. MLP networks have strong non-linear modeling capabilities and can perform complex feature extraction and non-linear modeling through multiple non-linear layers, making them well-suited for handling non-linear problems. The two models are defined as Model_SVM_ and Model_MLP_, and ${\left\{\text{B}-V_{i}\right\}}_{i=1}^{N}$of a subject are represented as *F* (i.e., Eq. 12), while the classification process of the Model_SVM_ and Model_MLP_ for **F** can be represented as Eqs 13, 14:


(12) $$\text{F}=\left[\text{B}-V_{1},\text{B}-V_{2},\ldots ,\text{B}-V_{N}\right]$$

(13) $$\hat{y}={\text{Model}}_{\text{SVM}}\,\left(F\right)$$

(14) $$\hat{y}={\text{Model}}_{\text{MLP}}\,\left(F\right)$$

**FIGURE 5:**
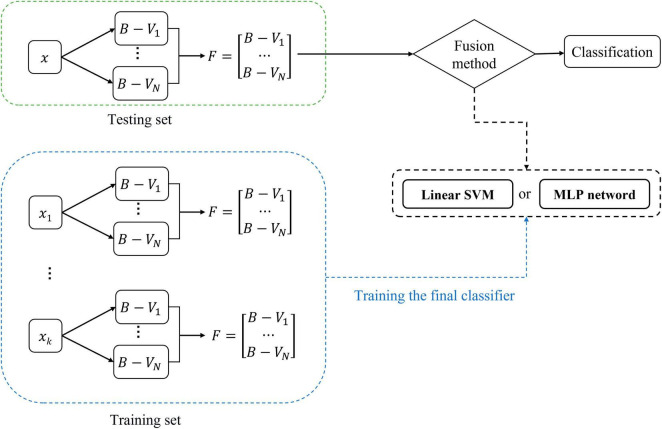
Training and classification of fusion methods.

Figure 5 shows the training and classification processes of the fusion method. Model_SVM_ and Model_MLP_ can first be trained on the training set and then evaluated on the testing set. The features used to train the classifier are the *F* of the subjects in the training set. In the testing phase, the **F** of a subject is input into the trained Model_SVM_ or Model_MLP_ to obtain its classification result.

## 3 Results

### 3.1 Experimental settings

Different features were extracted through a preprocessing process. In ROI selection, the optimal threshold for selecting features highly correlated with clinical status was determined from a set of five candidate *p*-values: 0.01, 0.02, 0.03, 0.04, and 0.05. The classification framework works on MATLAB, where the linear SVM was implemented using the LIBSVM package (Chang and Lin, 2011). The MLP network has two hidden layers, each containing five neurons, using the “tansig” function as the activation function for each hidden layer and mean-square error (MSE) as the loss function. In the experiment, ASD and NC were defined as negative and positive samples, respectively.

We used cross-validation to evaluate the performance of the proposed method. Particularly, feature selection and parameter optimization were performed on the training set only by internal cross-validation to ensure that the whole process ran automatically and also to avoid positively biased performance evaluation. The internal cross-validation for the most discriminative ROI and the optimal parameters determination (i.e., the parameters of the SVM) ensured the generalization of the proposed classification framework. All experiments were evaluated 10 times by 10-fold cross-validation, with the process being repeated 10 times to avoid the deviation of random data division in cross-validation. Specifically, all data were divided into 10 subsets of the same size, with 1 part of each subset serving as the testing set and the other 9 parts serving as the training set. In order to avoid any possible bias in the fold selection, the whole 10-fold cross-validation process was repeated 10 times, each time with a different random division of the samples. It should be noted that the hyperparameters in the “ROI selection, SVM training” process were based on the training subjects and were tuned by nested 10-fold cross-validation to avoid the effect of overfitting.

### 3.2 Classification performance

To evaluate the proposed classification framework, the accuracy (ACC), sensitivity (SEN), specificity (SPE), positive predictive value (PPV), negative predictive value (NPV), and F1 score, were calculated from the classification confusion matrix. Moreover, the *p*-values of the proposed method and the comparison methods were given.

### 3.3 Performance of classification framework

To evaluate the performance differences between the proposed classification framework with a single classifier, we performed experiments using 10 times of 10-fold cross-validation. First, a number of independent SVM of *N* was set for each feature in ${\left\{\lambda_{i}\right\}}_{i=1}^{N}$. The *p*-value and the optimal parameters of the SVM were obtained by internal cross-validation. After that, B−V was calculated for each classifier (i.e., SVM) based on the selected parameters, and the classification result of each classifier was derived based on Eq. 9. Meanwhile, the performance evaluation of all single classifiers was obtained. Furthermore, we fused the B−Vs derived from the classifier by using each of the three fusion methods yielding three fusion results, which were represented as B−V with linear SVM, B−V with majority voting, and B−V with MLP. The classification performances of the proposed multi-classifier fusion classification framework and all single classifiers are shown in Table 2. The best results are highlighted in bold.

**TABLE 2:** Performance statistics.

Method	ACC (%)	SEN (%)	SPE (%)	PPV (%)	NPV (%)	*p*-Values
Kurtosis	61.75 ± 0.30	62.23 ± 0.25	61.98 ± 0.49	68.12 ± 0.36	62.83 ± 0.31	0.012
VMD	63.94 ± 0.18	66.71 ± 0.22	64.87 ± 0.31	61.97 ± 0.11	65.62 ± 0.35	0.021
Mean	66.02 ± 0.45	72.45 ± 0.44	60.45 ± 0.42	72.55 ± 0.34	66.46 ± 0.51	0.013
Variance	69.39 ± 0.12	70.67 ± 0.33	57.50 ± 0.44	63.67 ± 0.14	67.33 ± 0.21	0.021
Skewness	70.81 ± 0.25	63.77 ± 0.39	71.56 ± 0.34	64.71 ± 0.25	71.34 ± 0.04	0.015
Sample entropy	71.05 ± 0.49	73.52 ± 0.14	68.53 ± 0.21	74.54 ± 0.02	70.14 ± 0.39	0.017
ALFF	74.51 ± 0.13	74.72 ± 0.23	74.43 ± 0.31	76.51 ± 0.45	75.75 ± 0.21	0.012
B-V with linear SVM	77.65 ± 0.19	74.12 ± 0.12	73.35 ± 0.18	77.91 ± 0.50	76.16 ± 0.11	0.014
B-V with majority voting	75.92 ± 0.34	68.13 ± 0.21	74.02 ± 0.11	68.92 ± 0.11	77.21 ± 0.15	0.011
B-V with MLP (ours)	**81.26 ± 0.23**	**78.50 ± 0.34**	**79.50 ± 0.55**	**78.98 ± 0.24**	**79.14 ± 0.12**	–

The experimental results in Table 2 show that (1) the single classifier with ALFF showed the highest performance among all other single classifiers with an accuracy of 74.51%, (2) the classification performance in the three classification frameworks with different fusion methods (i.e., B−V with linear SVM, B−V with majority voting, and B−V with MLP) outperformed any single classifier and the accuracy of the best-performing classification framework (i.e., B−V with MLP) outperformed the best performing single classifier (i.e., ALFF) by 5.55%, and (3) among the classification frameworks that used different fusion methods, the fusion method using MLP networks had the best performance, outperforming linear SVM and majority voting.

Based on Table 2, we can conclude that (1) the fusion method can effectively fuse multiple B−Vs and reduce the influence of unreliable B−Vs, which can improve the accuracy and reliability of the classification framework, and (2) in the fusion method, majority voting treats all classifiers equally, becoming unable to measure the weights of different classifiers, and linear SVM can only linearly fuse the B−Vs. In contrast, the fusion method with the MLP network as the classification framework measures the weights of different classifiers through the B−V on the one hand and captures the non-linear relationship between the B−Vs through the non-linear fitting ability of the MLP network on the other hand.

## 4 Discussion

### 4.1 Analysis of discriminative ROIs

**FIGURE 6:**
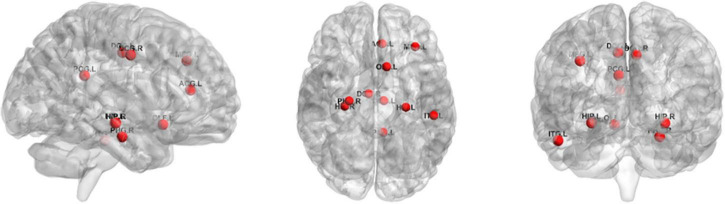
The top 10 ROIs via ROI selection.

For the ROI selection, we computed the frequency of each ROI in cross-validation (frequency was defined as the ratio of brain regions occurring in cross-validation) and selected 10 ROIs with the highest frequency of occurrence as the most discriminative ROIs. The top 10 ROIs were MFG.L, OLF.L, ACG.L, DCG.L, DCG.R, PCG.L, HIP.L, HIP.R, PHG.R, and ITG.L. Figure 6 illustrates these ROIs. As can be seen from the results, the discriminatory ROIs selected were in general agreement with the results reported in previous ASD studies (Chandana et al., 2005; Jin et al., 2015).

### 4.2 Ablation study for belief-value

To better understand the role of the output probability of SVM, *s_j_*, and *w_j_* in the classification framework, we set up two different forms of B−V computed by *f_j_*, *f*_*j*_ = *s*_*j*_ and *f*_*j*_ = *w*_*j*_, respectively, and compared them with the classification framework of B−V computed according to Eq. 8. B−V by *s_j_* and B−V by *w_j_* indicate that the information from the perspective of distance and the information from the perspective of local density were used, respectively. B−V by *w*_*j*_ × *s*_*j*_ indicates that the information from both perspectives was used. Table 3 shows the performance of the classification framework based on three different B-V, where B-V by *w*_*j*_ × *s*_*j*_ performed best and B-V by *s_j_* outperformed B-V by *w_j_*. The best results are highlighted in bold.

**TABLE 3:** Performance comparison of classification frameworks with different B-V.

Method	ACC (%)	SEN (%)	SPE (%)	PPV (%)	NPV (%)	*p*-Values
B−V by *w_j_*	74.67 ± 0.28	73.51 ± 0.45	68.53 ± 0.41	74.55 ± 0.23	72.61 ± 0.23	0.014
B−V by *s_j_*	76.56 ± 0.51	72.21 ± 0.33	76.51 ± 0.16	76.33 ± 0.43	73.69 ± 0.12	0.012
B−V by *w*_*j*_×*s*_*j*_ (ours)	**81.26 ± 0.23**	**78.50 ± 0.34**	**79.50 ± 0.55**	**78.98 ± 0.24**	**79.14 ± 0.12**	–

Based on the experimental results in Table 3, it can be concluded that (1) B−V, which effectively combines distance information and local density information, had a better ability to measure the degree of belief, and the proposed DST method was effective in converting information from two different perspectives, and (2) B−V, which considers only distance information, had a better ability to measure credibility than B−V, which considers only local density information, and distance information had more discriminative power than local density information.

### 4.3 The effect of distance metric for belief-value

To investigate the effect of B−V with different metrics of distance on the performance of the classification framework, we performed experiments based on three metrics of distance: Euclidean distance, Mahalanobis distance, and Manhattan distance. The MLP network was selected as the fusion method. The experiment results are shown in Table 4, and the best results are marked in bold.

**TABLE 4:** Performance of different distance metric.

Metric	ACC (%)	SEN (%)	SPE (%)	PPV (%)	NPV (%)	*p*-Values
Euclidean	78.14 ± 0.22	75.67 ± 0.12	75.39 ± 0.19	77.17 ± 0.21	72.62 ± 0.12	0.012
Manhattan	79.69 ± 0.33	72.50 ± 0.26	74.45 ± 0.37	72.67 ± 0.31	77.94 ± 0.43	0.016
Mahalanobis (ours)	**81.26 ± 0.23**	**78.50 ± 0.34**	**79.50 ± 0.55**	**78.98 ± 0.24**	**79.14 ± 0.12**	–

Table 4 shows that the Mahalanobis distance worked best as the metric of distance for belief-value. Since the Mahalanobis distance was not affected by the dimension, the Mahalanobis distance between two points was independent of the measurement unit of the original data, and it could also exclude the interference of correlation between variables, so it achieved better results than the Euclidean distance and Manhattan distance.

### 4.4 Results on validation datasets

To validate the robustness of our proposed method, we conducted experiments on new real multi-site ASD datasets of four imaging sites (Leuven, UCLA, UM, and USM). References for information about the dataset are given in the literature (Wang et al., 2019). The preprocessing procedure is the same as that mentioned in section “2.2. Data preprocessing.” We validated the proposed B-V with the MLP method on a multi-site dataset by 10 times of 10-fold cross-validation.

Table 5 shows the single-classifier performance for the multi-classifier fusion method and the optimal performance evaluated on each site dataset. The best results are highlighted in bold. The experimental results showed that (1) ALFF achieved the best single classifier performance on all site datasets, which indicates that ALFF is the effective classification feature for ASD diagnosis, and (2) the proposed multi-classifier fusion method achieved better classification performance than the optimal single classifier performance on all site datasets, which further demonstrates the effectiveness of multi-classifier fusion.

**TABLE 5:** Performance in different sites datasets.

Target site	Method	ACC (%)	SEN (%)	SPE (%)	PPV (%)	NPV (%)	*p*-Values
Leuven	ALFF	71.34 ± 0.21	70.42 ± 0.12	71.27 ± 0.24	70.06 ± 0.14	69.76 ± 0.37	0.015
	B-V with MLP	**78.68 ± 0.15**	**75.67 ± 0.26**	**72.83 ± 0.11**	**74.13 ± 0.32**	**73.91 ± 0.21**	–
UM	ALFF	73.09 ± 0.14	74.21 ± 0.19	72.15 ± 0.19	71.14 ± 0.44	72.16 ± 0.29	0.012
	B-V with MLP	**79.06 ± 0.23**	**74.39 ± 0.13**	**74.16 ± 0.14**	**72.01 ± 0.23**	**74.32 ± 0.13**	–
UCLA	ALFF	73.11 ± 0.19	72.19 ± 0.32	73.42 ± 0.25	75.24 ± 0.34	74.55 ± 0.17	0.011
	B-V with MLP	**80.54 ± 0.13**	**74.09 ± 0.21**	**73.67 ± 0.18**	**77.24 ± 0.14**	**75.31 ± 0.18**	–
USM	ALFF	73.23 ± 0.11	74.67 ± 0.23	72.21 ± 0.31	74.23 ± 0.45	72.26 ± 0.21	0.013
	B-V with MLP	**81.02 ± 0.12**	**77.61 ± 0.14**	**74.27 ± 0.22**	**74.34 ± 0.13**	**75.16 ± 0.25**	–

## 5 Conclusion

This study proposes a new belief-value and captures the non-linear relationship between belief-values from multiple classifiers through the MLP network, thus achieving better multi-classifier fusion. The experimental results have shown that (1) the representation of belief-value and NLP networks as fusion methods are reasonable and greatly improve the diagnostic performance, and (2) the representation of belief-value is enhanced by the DST method by using distance information and local density information. In general, our multi-classifier fusion classification framework is effective and outperforms the single-classifier method.

Finally, it should be noted that the use of local density information is not only possible in combination with SVM, but its use in other classifiers deserves to be explored, which will be the focus of our future research work.

## Data availability statement

Publicly available datasets were analyzed in this study. This data can be found here: https://fcon_1000.projects.nitrc.org/indi/abide/.

## Author contributions

FZ: Conceptualization, Methodology, Writing – review and editing. SY: Conceptualization, Formal analysis, Investigation, Methodology, Software, Validation, Writing – original draft. MLZ: Validation, Writing – review and editing. KL: Writing – review and editing. XQ: Writing – review and editing. YL: Writing – review and editing. NM: Writing – review and editing. YR: Writing – review and editing. MYZ: Writing – review and editing.
